# A Rare Case of Prostatic Stromal Tumour of Uncertain Malignant Potential Surrounding Ejaculatory Ducts in a Patient With Concurrent Prostate Adenocarcinoma

**DOI:** 10.7759/cureus.31690

**Published:** 2022-11-20

**Authors:** Kimberley Chan, John Piedad, Amal Mudiyanselage, Husay Janebdar, Ashish Narula, Freddie Banks, Jacques Roux, Nikhil Vasdev

**Affiliations:** 1 Urology, East and North Hertfordshire NHS (National Health Service) Trust, Stevenage, GBR; 2 Pathology, East and North Hertfordshire NHS (National Health Service) Trust, Stevenage, GBR; 3 Urology, West Hertfordshire Teaching Hospitals NHS (National Health Service) Trust, Watford, GBR

**Keywords:** prostate adenocarcinoma, stromal tumour of uncertain malignant potential, uncertain malignant potential, prostate stromal tumour, stump

## Abstract

Stromal tumour of uncertain malignant potential (STUMP) is exceedingly rare. Diagnosis and management of STUMP present a challenge to the urologist due to the absence of specific clinical findings and its unpredictable clinical course. Thus, radical resection is often recommended. Here, we present a case of a 64-year-old male, who presented with mild obstructive voiding symptoms with a raised age-specific prostate-specific antigen (PSA) of 3.1. Magnetic resonance imaging (MRI) showed an area of suspicion, in an area thought to be the left seminal vesicle, containing a malignant lesion within it. Biopsy of this area and the prostate confirmed concurrent prostatic STUMP and Gleason 3+3=6 adenocarcinoma of the prostate, managed with robotic-assisted laparoscopic radical prostatectomy with wide local excision.

## Introduction

Prostatic stromal tumour of uncertain malignant potential (STUMP) arises from mesenchymal tissue and is a rare entity, accounting for <1% of prostate cancer [1]. It is characterised by atypical stromal proliferation and exhibits an unpredictable clinical course, ranging from incidental finding to infiltration of neighbouring structures, to distant metastasis and even death [2-5]. Surgical excision is often warranted. Diagnosis of STUMP is challenging as clinical signs, symptoms, and laboratory findings associated with STUMP are non-specific, often resulting in diagnostic delay. 

Here, we present a 64-year-old patient who presented with urinary frequency and had raised age-specific prostate-specific antigen (PSA) of 3.1. This case is unique as only a few cases of concurrent prostatic adenocarcinoma and STUMP have been described in the literature, and decision-making surrounding its treatment remains poorly understood.

## Case presentation

A 64-year-old male initially presented to his general practitioner (GP) with increased urinary frequency. There was no history of urinary tract infection, haematuria, or haematospermia. He had a history of erectile dysfunction but was otherwise fit and well and did not take any regular medications. There was a history of prostate cancer with his father and breast cancer with his sister. He was a non-smoker, drank occasional alcohol, and worked in the IT sector. 

**Figure 1:**
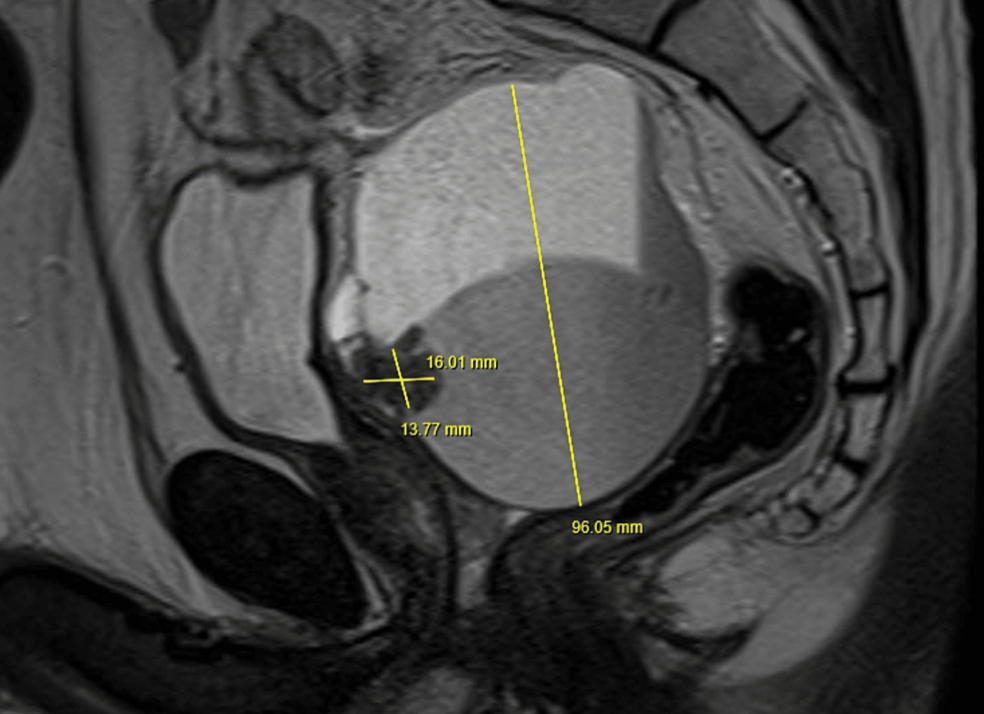
MRI pelvis-prostate Dilated haemorrhagic left seminal vesicle measuring 9.6cm with a hypercellular lesion (13.8 x 16mm) within, located superior to the prostatic base

He had an enlarged prostate with a craggy surface on digital rectal examination. He had a raised age-specific PSA of 3.1ng/ml, which triggered a two-week wait referral from the GP to the Urologist. A subsequent MRI pelvis prostate identified no target lesion within a 34cc prostate (PSA density 0.1). Interestingly, it identified what was assumed to be a 9.6cm dilated haemorrhagic left seminal vesicle (SV) containing haemorrhage of deferring ages, with a hypercellular lesion measuring 13.8x16.0mm within (Figure 1), located superior to the prostatic base. A rigid cystoscopy to visualise the trigone showed a normal urethra and ureteric orifices with no mucosal abnormalities. The patient underwent a transperineal template biopsy of the prostate and SV, which identified Gleason 3+3=6 adenocarcinoma of the prostate in 2/20 cores and STUMP within the left SV.

A multidisciplinary team (MDT) discussed this patient’s case and recommended a radical robotic-assisted laparoscopic prostatectomy (RALP) with wide local excision. The procedure was successfully performed with the da Vinci® Xi robotic platform (Intuitive Surgical, California, United States) with a total operative time of 180 minutes, encompassing 160 minutes of console time. Intra-operatively, the SVs were found to be large and tethered but removed en bloc with the prostate. He had a successful trial without a catheter on postoperative day 12.

**Figure 2:**
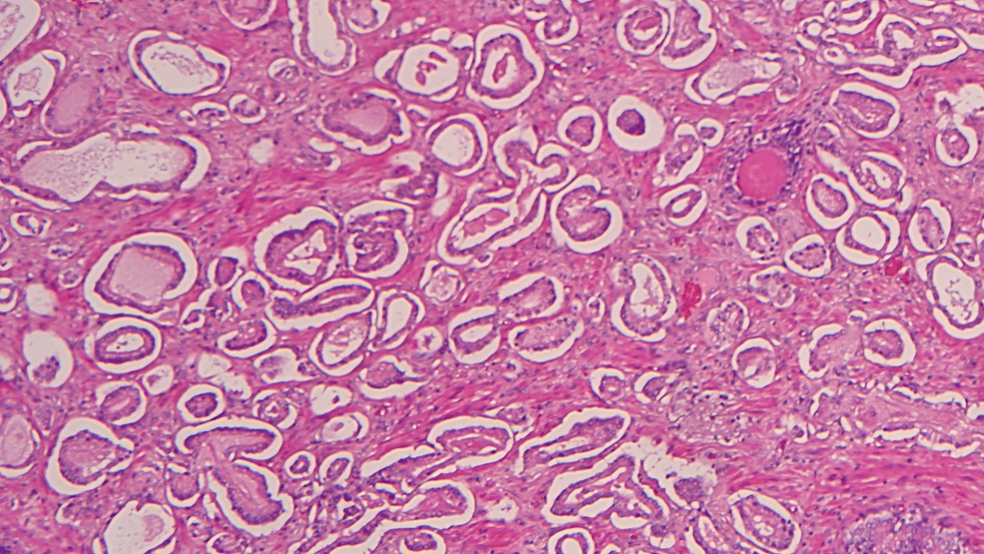
Microacinar adenocarcinoma of prostate (Haematoxylin and eosin stain x10)

The final histopathological report showed a pT2a R0 Gleason 3+3=6 microacinar adenocarcinoma of the prostate on the left side with negative resection margins and normal SVs (Figure 2). 

**Figure 3:**
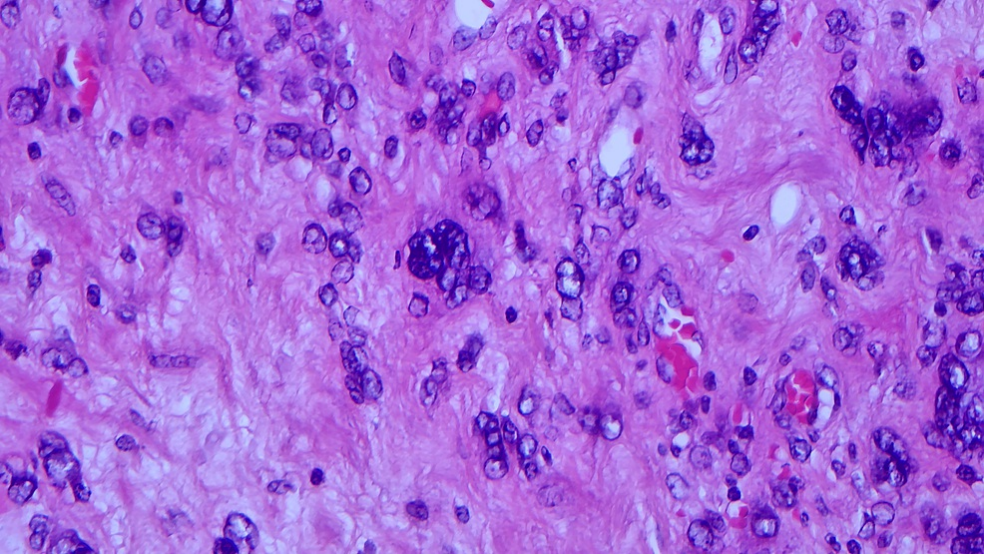
Atypical and multinucleated cells in stroma (Haematoxylin and eosin stain x40)

The maximum linear dimension of the dominant tumour nodule was relatively small at 12mm. There was a cyst on the posterior aspect of the prostate gland, adherent to the seminal vesicles anteriorly, surrounding the ejaculatory ducts. The cyst was lined by prostatic-type epithelium and the stroma surrounding the cyst showed atypical, pleomorphic, and multinucleated cells (Figure 3). Resection margins for the cyst were clear. The cellular stroma showed degenerative atypia, reminiscent of a phyllodes-like stromal proliferation described in the breast. Mitosis and necrosis were not identified. Immunohistochemical analysis was consistent with STUMP, from strong positive staining for vimentin, desmin, androgen, and progesterone receptors; moderate positive staining for oestrogen receptor; and negative staining for PSA and PanCK markers. Ki67 was low (<10%).

The patient had a follow-up PSA three months after RALP, which was <0.03ng/ml. The MDT recommended following the patient up with regular PSA checks and imaging, with the first follow-up MRI scan booked for six months post RALP.

## Discussion

STUMP is a non-epithelial, mesenchymal spindle-cell tumour that accounts for <1% of prostate cancer and only a few cases have been described in the literature [1]. They are considered neoplastic entities due to their diverse clinical behaviour. Some grow slowly over a period of several years, whereas others can infiltrate the prostate gland, invade adjacent tissue structures, recur after surgery, or progress to stromal sarcoma [2]. Three cases of distant metastasis have been reported in the literature and two patients in these reports died of their illnesses [3-5]. 

STUMP has been described in patients aged between 27 to 83 years old with a peak incidence in the sixth and seventh decade [2,6]. A clinicopathological study of 36 patients with STUMP identified obstructive urinary symptoms and abnormal digital rectal examination as the most common presenting symptom and examination findings, respectively [2]. Other clinical presentations described include haematuria, haematospermia, or a palpable rectal mass [2,6]. An elevated PSA is often a motivating factor for further urological investigations and prostate biopsy, leading to their diagnosis. However, there is no consensus on the relationship between PSA values and the tumour type and extent [2,7]. Diagnosis of STUMP can only be made histopathologically.

The current World Health Organisation (WHO) classification includes two diagnostic categories for mesenchymal tumours unique to the prostate: stromal sarcoma and STUMP [8]. The term ‘STUMP’ was used after Gaudin et al. studied the clinicopathological features of 18 patients with STUMP and described four histological patterns including phyllodes-like, myxoid spindle cells, hypercellular spindle cells, and degenerative atypia [6]. Another recently described histology had a novel round-cell pattern [7]. It is important to distinguish STUMP from stromal sarcoma as STUMP can occur by itself or be associated with stromal sarcoma either concurrently or subsequently [2], with the latter associated with poor prognosis [9]. STUMP is characterised by the presence of large, bizarre cells with vacuolated nuclei and frequent multinucleation. A study on 22 patients with either prostatic stroma sarcoma (PSS) or STUMP identified greater cellularity, mitotic figures, necrosis, and stromal overgrowth in patients with PSS.

Immunohistochemical studies on patients with STUMP and PSS showed positivity for CD34 and progesterone receptors but no activity for oestrogen receptors. Desmin, smooth muscle actin, and HHF-35 were positive in 80%, 40%, and 50% of patients with STUMP, respectively, but this was absent in PSS patients. The expression of muscle markers in STUMP, therefore, provides a function for differentiating them from PSS. Furthermore, the Ki67 index is typically low in STUMP but high in PSS. In our case, the absence of mitosis and necrosis in the cellular stroma, low Ki67 index (<10%), and strong positivity for progesterone and desmin favour STUMP.

There are no guidelines for the treatment of STUMP due to its rarity. Management of STUMP is challenging due to its diverse clinical behaviour, with the potential of progressing to stromal sarcoma and metastasis. Thus, radical resection is often recommended. Active surveillance has also been described in the literature. A case series on 14 STUMP patients who had active surveillance post biopsy or transurethral resection reported no disease progression after an average follow-up of 4.9 years [2]. Ultimately, decisions on treatment options should involve an MDT and be tailored on an individual basis. An intervention should be pursued only after a thorough discussion of the risk and benefits of intervention with the patient. In our case, we recommended radical resection in the form of RALP for STUMP and regular follow-up to monitor for recurrence, in the form of PSA and MRI pelvis-prostate imaging.

## Conclusions

STUMP is a rare entity, with only a few cases of concurrent STUMP and prostatic adenocarcinoma described in the literature. Diagnosis of STUMP is difficult due to the absence of specific clinical and laboratory findings. Management of STUMP presents challenge to the urologist due to its diverse and unpredictable clinical course, from being indolent to potential of progressing to stromal sarcoma and distant metastasis. There are no universal guidance on the management of STUMP due to its rarity but we recommend radical resection in the form of RALP in cases of concurrent STUMP and prostatic adenocarcinoma, with regular follow-up in the form of serial PSAs and MRI pelvis-prostate imaging. 
